# Supplementary material for: Evidence supported by Mendelian randomization: impact on inflammatory factors in knee osteoarthritis
Source: Front Med (Lausanne). 2024 May 28;11:1382836. doi: 10.3389/fmed.2024.1382836 (PMC11165061; doi:10.3389/fmed.2024.1382836)
Supplement: Supplementary file 1 [file Data_Sheet_1.ZIP › data/GCST90274773_5e6/773.forest.pdf]

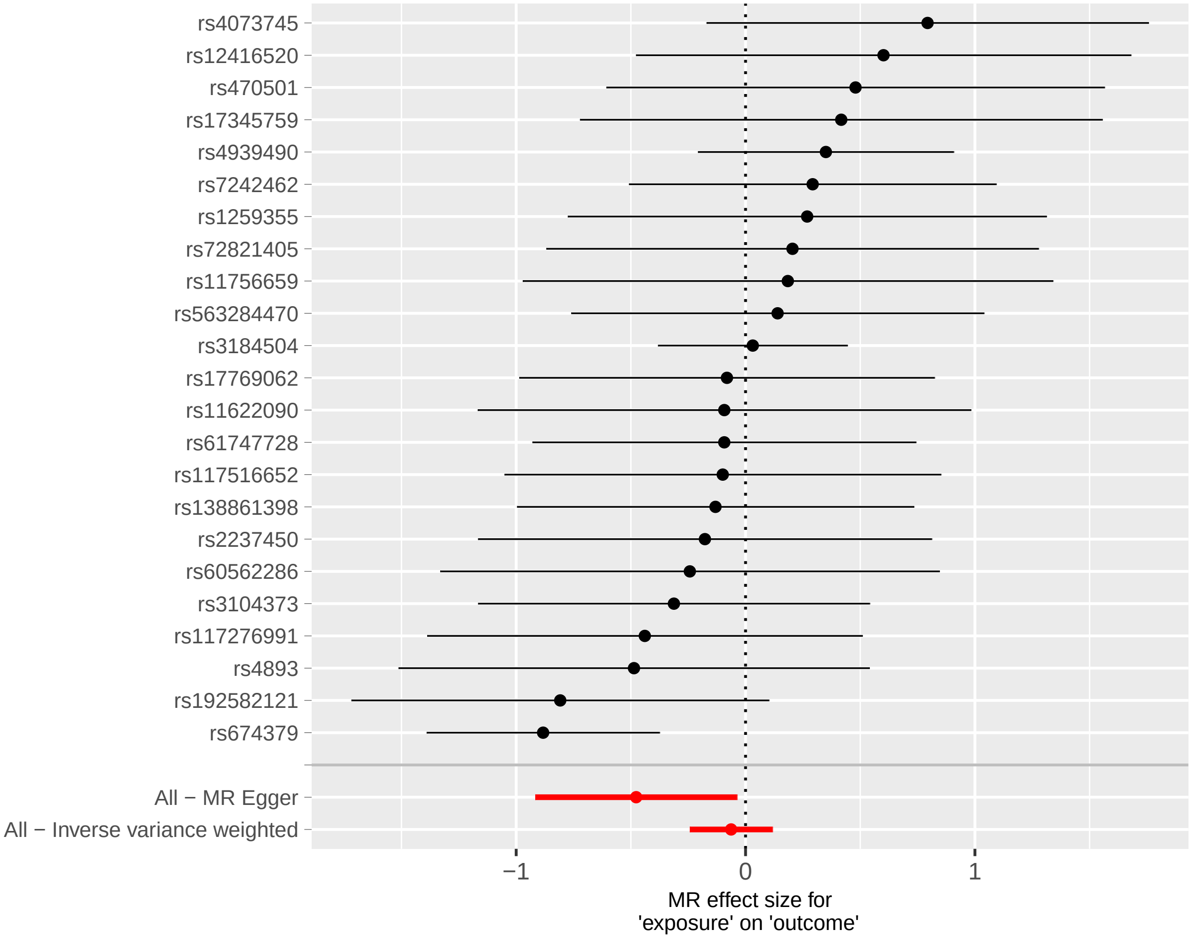

rs4073745
rs12416520
rs470501
rs17345759
rs4939490
rs7242462
rs1259355
rs72821405
rs11756659
rs563284470
rs3184504
rs17769062
rs11622090
rs61747728
rs117516652
rs138861398
rs2237450
rs60562286
rs3104373
rs117276991
rs4893
rs192582121
rs674379
All − MR Egger
All − Inverse variance weighted
−1
0
1
MR effect size for
'exposure' on 'outcome'
